# Systolic anterior motion of the mitral valve associated with acute type A aortic dissection: a case report describing one-step surgery of ascending aortic replacement and interventricular septal myectomy

**DOI:** 10.1186/s40792-021-01268-7

**Published:** 2021-08-16

**Authors:** Naoki Yamamoto, Koji Onoda

**Affiliations:** Department of Cardiovascular Surgery, Shingu Municipal Medical Center, 18-7 Hachibuse, Shingu, Wakayama 647-0072 Japan

**Keywords:** Ascending aortic dissection, Interventricular septal myectomy, Relative adrenal failure, Systolic anterior motion

## Abstract

**Background:**

Systolic anterior motion of the mitral valve associated with acute type A aortic dissection is rare in daily clinical practice. The prevention of systolic anterior motion is important, because once it occurs, the hemodynamics may become unstable, leading to a critical situation. In the surgical procedure to treat systolic anterior motion, the prevention of new iatrogenic aortic intimal tears is important in the context of acute type A aortic dissection.

**Case presentation:**

We present a case of systolic anterior motion in a 68-year-old woman with an acute type A aortic dissection and suspected acute relative adrenal insufficiency. Preoperative transthoracic echocardiography revealed left ventricular outflow tract obstruction due to systolic anterior motion without left ventricular hypertrophy and interventricular septal bulging due to a narrow aorto-mitral angle. We successfully performed a one-step surgery for ascending aortic replacement and interventricular septal myectomy using the needle stick technique for the treatment of systolic anterior motion.

**Conclusions:**

Concomitant interventricular septal myectomy using the needle stick technique with thoracic aortic replacement is a safe and feasible technique. Interventricular septal myectomy may be effective in preventing postoperative unstable hemodynamics due to systolic anterior motion in the management of acute aortic dissection.

## Background

Systolic anterior motion (SAM) of the mitral valve is a cardiac functional disorder that can be induced by multifactorial dynamic components, such as left ventricular (LV) structures, mitral valvular or subvalvular structures, and patients’ cardiovascular hemodynamics [1–4]. Preventing SAM is important, because once SAM occurs, the hemodynamics may be unstable, leading to a critical situation [5]. Here, we present a rare case of a patient with unstable SAM associated with acute type A aortic dissection (AAD) who underwent a one-step surgery for ascending aortic replacement and concomitant interventricular septal myectomy (IVSM) for the treatment of SAM.

## Case presentation

**Fig. 1:**
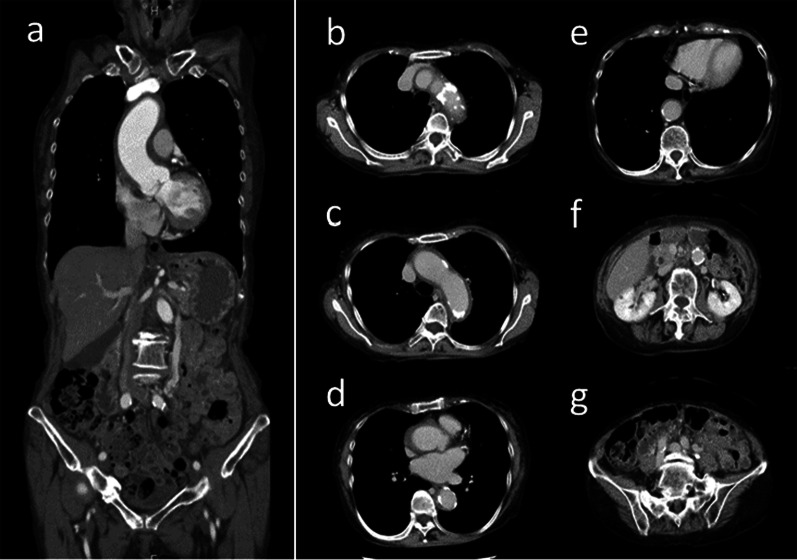
Computed tomography imaging of acute type A aortic dissection. **a** Dissected ascending aorta. The maximum short diameter of the ascending aorta was 39 mm, and the maximum thickness of the thrombosed false lumen was 4 mm. **b, c** Dissected aortic arch. **d** Dissected aortic root and descending aorta of left atrial level. **e** Dissected descending aorta. **f** Non-dissected infrarenal aorta. **g** Non-dissected iliac arteries

A 68-year-old woman (body weight, 48 kg; height, 158 cm) was referred to our emergency department for acute-onset chest and back pain. On arrival, her vital signs were as follows: respiratory rate, 17 breaths/min; temperature, 36.4 °C; blood pressure, 94/56 mmHg; heart rate, 73 beats/min; oxygen saturation level, 99% in ambient air; and Glasgow Coma Scale score 15. Physical examination revealed no heart murmur and normal respiratory sounds. A 12-lead electrocardiogram showed no evidence of acute coronary syndrome. Her medical history included Stanford type B aortic dissection, hypertension, and rheumatoid arthritis controlled with anti-rheumatic drugs and alternate-day oral corticosteroids (prednisolone 5 mg per dose). Computed tomography (CT) revealed thrombosed AAD from the aortic root to the renal arteries, no aortic rupture, and no malperfusion. The ascending aortic maximum short diameter was 39 mm, and the maximum thickness of the thrombosed false lumen was 4 mm (Fig. 1). Transthoracic echocardiography (TTE) revealed no evidence of cardiac tamponade, aortic valve insufficiency, or other significant valve diseases. Her vital signs were stable; therefore, an urgent operation at a later date for AAD was planned. Preoperative blood pressure control was performed in our intensive care unit, in combination with bed rest, to prevent recurrent aortic events. Her systolic blood pressure was maintained between 90 and 110 mmHg with continuous infusion of a calcium antagonist (nicardipine 0.3–1.2 µg/kg/min). Her heart rate was between 80 and 100 beats/min, and oxygen saturation was 98% at 2 l/min oxygenation with a nasal cannula.

**Fig. 2:**
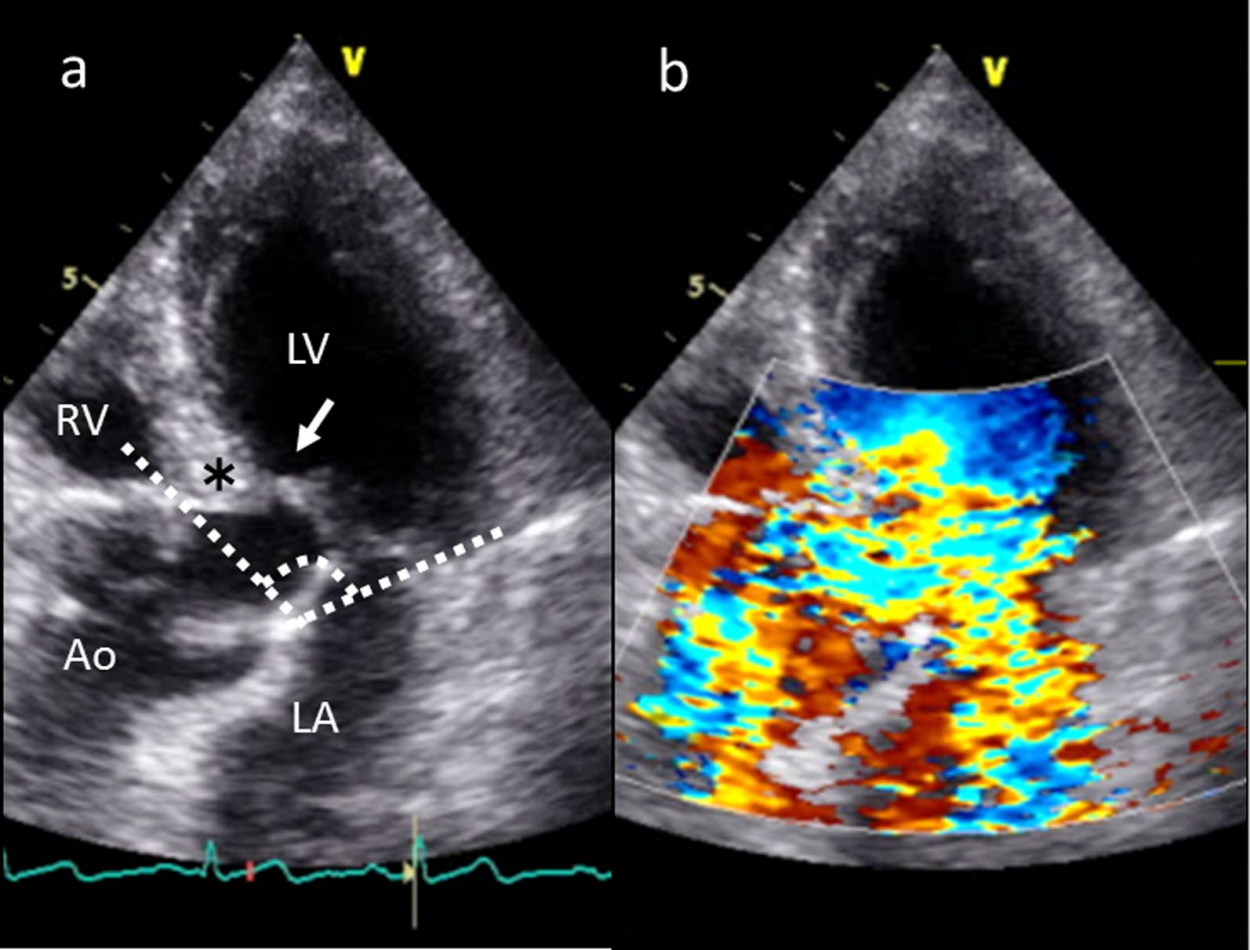
Preoperative transthoracic echocardiographic views. **a** Left ventricular outflow tract obstruction with systolic anterior motion of the mitral valve (white arrow), thick basal interventricular septum, and septal bulging (black asterisk) due to narrow aorto-mitral angle (dotted lines). **b** Color Doppler imaging of mitral regurgitation and flow acceleration in the left ventricular outflow tract. Ao: aorta; LA: left atrium; LV: left ventricle; RV: right ventricle

A day after the onset of aortic dissection, cardiac auscultation revealed a Levine 2/6 systolic murmur at the apex with no diastolic component. Subsequent investigation using TTE led to the detection of SAM of the mitral valve. TTE revealed LV hypovolemia, LV outflow tract (LVOT) obstruction due to SAM without LV hypertrophy (LVH), and interventricular septal (IVS) bulging due to a narrow aorto-mitral angle of 106.4° (Fig. 2a, b). The basal IVS thickness was 18.1 mm. The LV wall motion was hyperkinetic, with an ejection fraction of 81.7%. The peak flow velocity in the LVOT was 6.25 m/s. Abnormal laboratory data included hyponatremia (serum Na: 115.9 mEq/l), and her urine output per day was approximately 3000 ml. An adrenocorticotropic hormone stimulation test revealed suspected relative adrenal failure due to physical stress after acute aortic dissection. The systolic heart murmur due to SAM tended to improve with heart rate control to between 60 and 70 beats/min with beta-blocker therapy (landiolol hydrochloride 2 µg/kg/min) and infusion load.

**Fig. 3:**
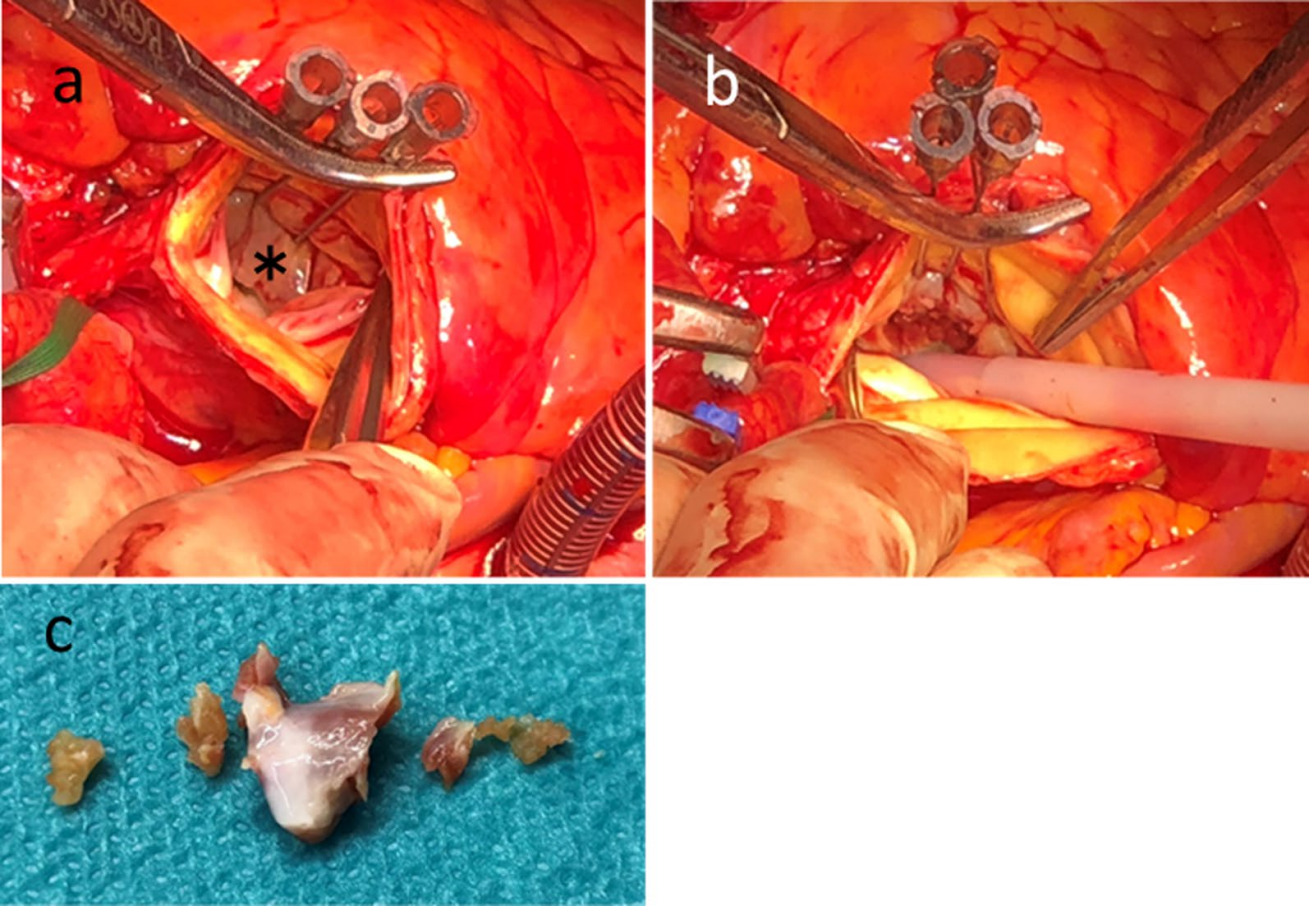
Needle stick technique of the interventricular septal muscle resection. **a** Exposure of the part of the interventricular muscle to be resected. Black asterisk indicates the interventricular septal bulge. **b** Resected part of the interventricular septal muscle. **c** Resected muscle pieces of 278 mg in total

**Fig. 4:**
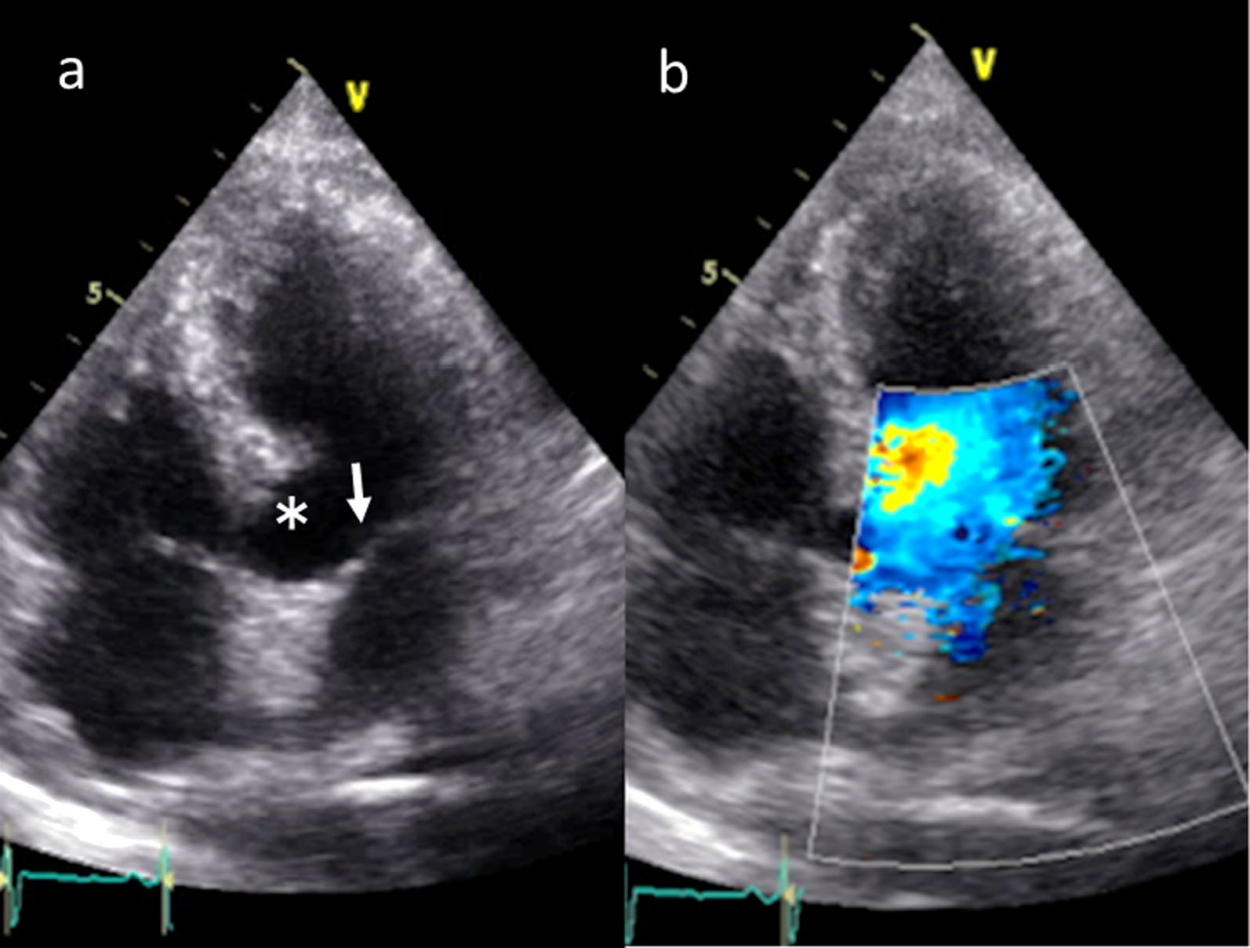
Postoperative transthoracic echocardiographic views. **a** No evidence of systolic anterior motion (white arrow) and resected part of the basal interventricular septum (white asterisk). **b** Color Doppler imaging of no mitral regurgitation

The aortic false lumen was still thrombosed, but the ascending aortic maximum short diameter was dilated from 39 to 45 mm and the maximum thickness of the thrombosed false lumen increased from 4 to 8 mm in the preoperative CT scan at 14 days after the onset of aortic dissection. In case of postoperative unstable hemodynamics following the physical stressful aortic repair, concomitant IVSM during the ascending aortic replacement was planned for the prevention of SAM. The patient’s adrenal insufficiency recovered gradually with alternate-day oral corticosteroid (prednisolone 5 mg per dose); therefore, we performed the operation 19 days after the onset of aortic dissection. In the operating room, transesophageal echocardiography under total anesthesia still showed SAM. Following median sternotomy, IVSM and ascending aortic replacement with a one-branched J Graft Shield Neo 26-mm Dacron graft (Japan Lifeline Inc., Tokyo, Japan) were performed under usual cardiopulmonary bypass with cannulation via the right femoral artery and right atrium. In the operative findings, there was no pericardial effusion that could compress the LV structure. During the IVSM procedure, the needle stick technique (NST) (Fig. 3a, b) was applied because of easy, quick, and protective exposure to the IVS muscle to be resected. Briefly, three 21-gauge needles were inserted into the interventricular septum just below the aortic valve annulus beyond the far side of the septal bulge. One needle was inserted beneath the mid portion of the right coronary cusp to provide a right-side margin, the second was inserted beneath the commissure of the right and left coronary cusps to provide a left-side margin, and the last was inserted between the previous two as a guide for the thickness of the myectomy (Fig. 3a). The resected muscle weighed 278 mg in total (Fig. 3c). The cardiopulmonary bypass was easily weaned. Postoperative TTE showed improved systolic flow velocity in the LVOT of 0.93 m/sec and no evidence of SAM (Fig. 4a, b). The patient was discharged from our hospital 22 days after the operation, with no major complications.

## Discussion

AAD is occasionally complicated by aortic and vascular events, such as aortic valve regurgitation, aortic rupture, and malperfusion, but cases of SAM complicating AAD have seldom been described [3]. The present case had unstable SAM induced anatomically and hemodynamically by AAD. The concomitant IVSM procedure, performed with NST during the ascending aortic replacement, was conducted to prevent unstable hemodynamics during postoperative management.

**Fig. 5:**
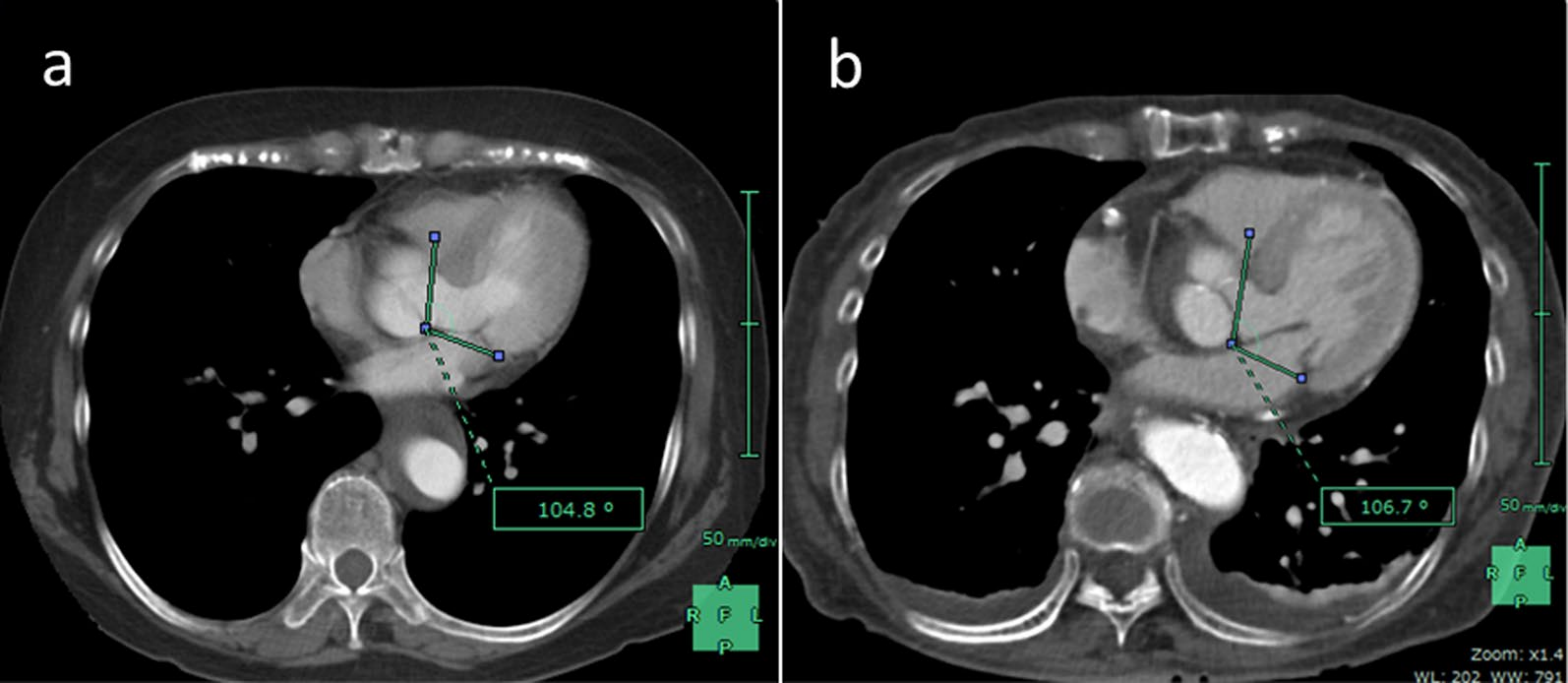
Narrow aorto-mitral angle detected in computed tomography. **a** Her past image before the onset of acute type A aortic dissection (at the onset of type B aortic dissection). **b** Preoperative image after the onset of acute type A aortic dissection. Both images have almost the same cross section

**Fig. 6:**
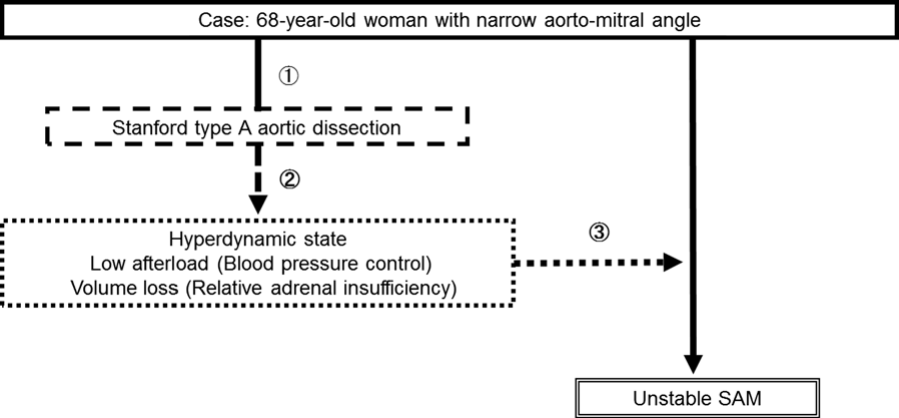
Possible mechanism of systolic anterior motion of the mitral valve associated with acute type A aortic dissection. **1** A 68-year-old woman with narrow aorto-mitral angle had acute type A aortic dissection. **2** Blood pressure control leading to low afterload was needed for prevention of recurrent aortic events, and physical stress due to aortic dissection caused relative adrenal insufficiency, leading to volume loss. **3** Unstable SAM of the patient with narrow aorto-mitral angle became apparent due to hyperdynamic state after aortic dissection, low afterload, and volume loss. *SAM* systolic anterior motion of mitral valve

SAM is a cardiac functional disorder to be avoided in perioperative cardiovascular management. SAM can be induced by dynamic interactions between cardiac anatomical features and hemodynamic conditions. The main mechanism of SAM has been recognized as the Venturi effect in the LVOT, and several cardiac anatomical features are known to affect the manifestation of SAM. SAM is most commonly seen in hypertrophic obstructive cardiomyopathy (HOCM). Structural abnormalities leading to SAM include LVH, a small LV cavity, IVS bulging due to a narrow aorto-mitral angle (< 120°), and a mismatch between the amount of mitral valvular tissue and the area of the mitral valve orifice [1–4]. Hemodynamic conditions leading to SAM include low systemic arterial resistance, hypovolemia, tachycardia, a hyperdynamic left ventricle, and cardiac tamponade [3, 4]. In addition, SAM may occur after aortic valve replacement and repair and mitral valve procedures such as mitral repair because of a discrepancy between the amount of leaflet tissue and the size of the annular ring selected in mitral annuloplasty [1, 2]. The anterior leaflet of the mitral valve exists on the LVOT in which the cardiac hemodynamics are finally concentrated. Therefore, SAM is considered a multifactorial cardiac functional disorder induced by the coexistence of several anatomical and hemodynamic components. The present case had, by chance, an anatomical feature of IVS bulging due to a narrow aorto-mitral angle before AAD. This is confirmed through a narrow aorto-mitral angle having been detected in her past CT images before the onset of AAD (Fig. 5a, b). Moreover, her medical history included rheumatoid arthritis controlled with oral corticosteroid medication. The SAM in the present case may have been affected by the patient’s specific cardiac structure of IVS bulging due to a narrow aorto-mitral angle, coupled with lower cardiac afterload due to blood pressure control to prevent recurrent aortic events, and hypovolemia due to the relative adrenal failure following aortic dissection (Fig. 6). Stable hemodynamics were not considered to be maintained by ascending aortic replacement alone because of reversible SAM in postoperative management. Therefore, concomitant IVSM during ascending aortic replacement was performed.

IVSM originates from the Morrow operation, which can diminish the Venturi effect with the resected IVS muscle in the LVOT [6]. IVSM has been conducted for the treatment of HOCM [6, 7], sigmoid or bulging septum, and LVH due to aortic stenosis and hypertension. Indeed, IVSM in itself has good clinical results; however, there are several possible methods for appropriately exposing the IVS muscle to be resected. Usui and colleagues developed the NST in the resection procedure of the IVS muscle and described a report of 17 cases other than aortic dissection [7]. The NST enables surgeons to get a good exposure of the IVS muscle and grasp the part of the muscle to be resected three-dimensionally. Unlike the aortic retractor, which carries the risk of aortic intimal injuries, the NST is helpful for surgeons to safely achieve sufficient IVSM even in the case of AAD.

## Conclusions

This report highlights the following points: (1) We presented a rare case of SAM associated with AAD that was cured by concomitant thoracic aortic replacement and IVSM. (2) We advocate the novel application of IVSM using the NST to cases of AAD. This is a safe and feasible procedure to prevent new intraoperative iatrogenic tears of the aortic intima and postoperative unstable SAM, even in the context of AAD.

## Data Availability

None.

## Abbreviations

SAM: Systolic anterior motion
LV: Left ventricular
AAD: Acute type A aortic dissection
IVSM: Interventricular septal myectomy
CT: Computed tomography
TTE: Transthoracic echocardiography
LVOT: Left ventricular outflow tract
LVH: Left ventricular hypertrophy
IVS: Interventricular septal
NST: Needle stick technique
HOCM: Hypertrophic obstructive cardiomyopathy
